# Origin and evolution of the peroxisomal proteome

**DOI:** 10.1186/1745-6150-1-8

**Published:** 2006-03-23

**Authors:** Toni Gabaldón, Berend Snel, Frank van Zimmeren, Wieger Hemrika, Henk Tabak, Martijn A Huynen

**Affiliations:** 1 CMBI, Center for Molecular and Biomolecular Informatics; NCMLS, Nijmegen Center for Molecular Life Sciences. Radboud University Nijmegen Medical Center. Toernooiveld 1. 6525 ED Nijmegen. The Netherlands; 2 ABC-Expression Centre, University of Utrecht, Padualaan 8, 3584 CX Utrecht, The Netherlands; 3 Laboratory of Cellular Protein Chemistry, University of Utrecht, Padualaan 8, 3584 CX Utrecht, The Netherlands; 4 Present address: Bioinformatics department, Centro de Investigación Principe Felipe. Avda. Autopista del Saler, 16. 46013 Valencia, Spain

## Abstract

**Background:**

Peroxisomes are ubiquitous eukaryotic organelles involved in various oxidative reactions. Their enzymatic content varies between species, but the presence of common protein import and organelle biogenesis systems support a single evolutionary origin. The precise scenario for this origin remains however to be established. The ability of peroxisomes to divide and import proteins post-translationally, just like mitochondria and chloroplasts, supports an endosymbiotic origin. However, this view has been challenged by recent discoveries that mutant, peroxisome-less cells restore peroxisomes upon introduction of the wild-type gene, and that peroxisomes are formed from the Endoplasmic Reticulum. The lack of a peroxisomal genome precludes the use of classical analyses, as those performed with mitochondria or chloroplasts, to settle the debate. We therefore conducted large-scale phylogenetic analyses of the yeast and rat peroxisomal proteomes.

**Results:**

Our results show that most peroxisomal proteins (39–58%) are of eukaryotic origin, comprising all proteins involved in organelle biogenesis or maintenance. A significant fraction (13–18%), consisting mainly of enzymes, has an alpha-proteobacterial origin and appears to be the result of the recruitment of proteins originally targeted to mitochondria. Consistent with the findings that peroxisomes are formed in the Endoplasmic Reticulum, we find that the most universally conserved Peroxisome biogenesis and maintenance proteins are homologous to proteins from the Endoplasmic Reticulum Assisted Decay pathway.

**Conclusion:**

Altogether our results indicate that the peroxisome does not have an endosymbiotic origin and that its proteins were recruited from pools existing within the primitive eukaryote. Moreover the reconstruction of primitive peroxisomal proteomes suggests that ontogenetically as well as phylogenetically, peroxisomes stem from the Endoplasmic Reticulum.

**Reviewers:**

This article was reviewed by Arcady Mushegian, Gáspár Jékely and John Logsdon

**Open peer review:**

Reviewed by Arcady Mushegian, Gáspar Jékely and John Logsdon. For the full reviews, please go to the Reviewers' comments section.

## Background

Peroxisomes were first isolated from liver and biochemically characterized by the group of de Duve [1]. Later it became clear that these organelles can differ substantially between species with respect to their enzyme content. The conversion of fatty acids into carbohydrates through the glyoxylate cycle is the hallmark of glyoxysomes present in plants, protozoa and yeasts. Part of the glycolysis is compartmentalized in the glycosomes of Trypanosomatids. Photorespiration is typical for plant peroxisomes while peroxisomes of various yeasts can oxidize alkanes or methanol. Despite this diversity all these organelles belong to the same microbody family. This became clear with the discovery that they share targeting codes (PTS1 and PTS2) for the import of proteins and with the identification of similar sets of proteins responsible for their biogenesis and maintenance [2]. Although the unity within the microbody family has thus firmly been established, their evolutionary origin remains a matter of debate [3]. Strong arguments support the view of peroxisomes as autonomous organelles with an endosymbiotic origin: i) matrix enzymes are synthesized on free polyribosomes and post-translationally imported into the organelles, ii) peroxisomes have their own protein import machinery, like mitochondria and chloroplasts, and iii) peroxisomes have been shown to divide [4].

Recent discoveries, however, have challenged this view. First, after several generations the lacking of peroxisomes in some mutants is reversible upon the introduction of the wild-type gene [5]. Second, it has been observed that new peroxisomes originate from the ER [6]. These observations are at odds with the autonomy of peroxisomes and therefore weaken the case for an endosymbiotic origin. Here we address the issue of peroxisomal evolution by phylogenetic analysis of peroxisomal proteins. To this end we collected an exhaustive set of proteins with an experimentally determined peroxisomal location in the yeast *Saccharomyces cerevisiae *and the rodent *Rattus norvegicus*, and performed phylogenetic analyses to investigate whether the peroxisomal proteome contains a significant evolutionary signal just as has been shown for mitochondria [7,8].

## Results and discussion

**Figure 1:**
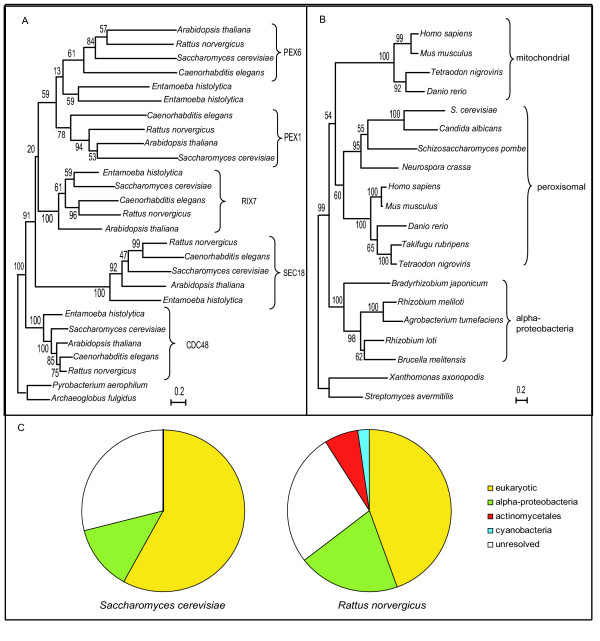
A: Maximum likelihood phylogenetic tree of the CDC48 orthologous group and its paralogs, including PEX1 and PEX6. The crenarchaeon *Pyrobaculum aerophilum *and euryarchaeon *Archaeoglobus fulgidus *sequences cluster together, consistent with an ancient eukaryotic origin of this protein family rather than an origin from a horizontal transfer, and they are used as outgroup. PEX1/6, as well as SEC18 and RIX7 appear to have evolved from CDC48, the central protein of the ERAD pathway B: Maximum likelihood phylogenetic tree of the Npy1p orthologous group and its mitochondrial paralogs. This protein family has a single origin in the alpha-proteobacteria. Bootstrap support over 100 replicates of the maximum likelihood tree is shown in all partitions. C: Pie chart showing the relative distribution of peroxisomal proteins according to their phylogenetic origin in yeast (left) and rat (right). Proteins that do have prokaryotic homologs but for which no reliable tree can de constructed, e.g. due to short stretches of homology, are considered "unresolved". For a complete list of the proteins and their origins, see the supplemental material, for their phylogenies see [44].

From databases and experimental literature we collected 62 yeast and 51 rat proteins with a peroxisomal location or function (Table 1). Since our lists include proteins from various large-scale proteomics analyses [9-11], as well as from individual studies under various conditions, we consider them to be representative samples of peroxisomal proteomes. Phylogenies (see materials and methods) of peroxisomal proteins were reconstructed to determine their origin. We consider a protein to be of eukaryotic origin when it has no homologs in prokaryotes, or when the prokaryotic branches within the tree are mono-phyletic as in Figure 1a. In the latter case the protein is classified as of "ancient origin" in Table 1, even though one could argue that in the case of PEX1 the protein resulted from a gene duplication at the origin of the eukaryotes. Although in the case of PEX1 the relatively short branch length of CDC48 suggests that CDC48 is the "ancestral protein" and PEX1 is derived, in general such a distinction is not easy to make and in this analysis we did not distinguish between genes that are, or are not duplicated at the origin of the eukaryotes. A protein is considered of bacterial or archaeal origin when it clusters "within" a prokaryotic branch, implying horizontal transfer between the taxa (Figure 1b). Unresolved cases imply the existence of homology to prokaryotic sequences without a tree that specifically supports a bacterial or archaeal origin. For the families with resolved phylogenies we observed a clear dichotomy in terms of evolutionary origin and functional roles: all proteins with a specific bacterial origin have enzymatic functions while most proteins (90%) with eukaryotic origin are functioning in peroxisome organization and biogenesis. Like in the proteins with bacterial ancestry also among the proteins with bacterial homologs for which we cannot establish bacterial ancestry (the unresolved cases) a clear preponderance (85%) of enzymes can be observed (Table 1).

**Table 1:** Proteins localized in the peroxisome in *S. cerevisiae *and *R. norvegicus*. Gene names are from SGD, Swissprot or GeneBank. Proteins in the same row are orthologous to each other, whenever there is a "one to many" orthology relationship this is indicated by boxes containing several rows. Absence of the gene or absence of evidence of a peroxisomal localization of the encoded protein is indicated by a dash. Proteins that show homology with components of the ERAD pathways are indicated with names in bold and a comment indicating that homology. For each orthologous group, the annotated function and the phylogenetic origin is indicated (euk: eukaryotic no bacterial homologs; euk (a.o.): presence of bacterial homologs but the phylogenetic reconsructions indicates an ancient origin derived from the common ancestor of eukaryotes and archaea; alpha: alpha-proteobacterial origin; actinomyc.: derived from the actinomycetales; cyanobac.: cyanobacterial origin; u, unresolved phylogenetic origin.

S. cerevisiae	R. norvegicus	Origin	Function (comment)
**PEX1**	**PEX1**	euk (a.o)	Peroxisome organization and biogenesis (Homologous to ERAD protein Cdc48)
**PEX2**	**PEX2**	euk	Peroxisome organization and biogenesis (Homologous to ERAD protein Hrd1)
PEX3	PEX3	euk	Peroxisome organization and biogenesis
**PEX4**	**PEX4**	Euk	Peroxisome organization and biogenesis (Homologous to ERAD protein Ubc1)
**PEX5**	**PEX5**	euk (a.o)	Peroxisome organization and biogenesis (Homologous to ERAD protein Hrd3)
PEX6	PEX6	euk (a.o)	Peroxisome organization and biogenesis
PEX7	PEX7	Euk	Peroxisome organization and biogenesis
PEX8	-	Euk	Peroxisome organization and biogenesis
**PEX10**	**PEX10**	Euk	Peroxisome organization and biogenesis (Homologous to ERAD protein Hrd1)
-	PEX11	euk (a.o)	Peroxisome organization and biogenesis
PEX12	PEX12	euk	Peroxisome organization and biogenesis
PEX13	PEX13	euk	Peroxisome organization and biogenesis
PEX14	PEX14	euk (a.o)	Peroxisome organization and biogenesis
PEX15	-	euk	Peroxisome organization and biogenesis
-	PEX16	euk	Peroxisome organization and biogenesis
PEX17	-	euk	Peroxisome organization and biogenesis
PEX18	-	euk	Peroxisome organization and biogenesis
PEX19	PEX19	euk	Peroxisome organization and biogenesis
PEX21	-	euk	Peroxisome organization and biogenesis
PEX22	-	euk	Peroxisome organization and biogenesis
PEX25	-	euk	Peroxisome organization and biogenesis
-	PEX26	euk	Peroxisome organization and biogenesis
PEX27	-	euk	Peroxisome organization and biogenesis
PEX28	-	euk	Peroxisome organization and biogenesis
PEX29	-	euk	Peroxisome organization and biogenesis
PEX30	-	euk	Peroxisome organization and biogenesis
PEX31	-	euk	Peroxisome organization and biogenesis
PEX32	-	euk	Peroxisome organization and biogenesis
ANT1	PMP34	euk	Adenine nucleotide transporter
-	PMP24	euk	Peroxisomal membrane protein
-	PMP22	euk	Peroxisomal membrane protein
-	PAHX	U	Phytanoyl-CoA dioxygenase
-	gi-6912418	U	2-hydroxyphytanoyl-CoA lyase
-	PTE2B	alpha	peroxisomal long chain acyl-CoA thioesterase Ib
TES1	PTE1_MOUSE	alpha	Peroxisomal acyl-coenzyme A thioester hydrolase 1
CTA1	CATALASE	euk (a.o)	Catalase A
FOX1	OXRTA2	U	acyl-CoA oxidase
gi-1684747	U		
CAO3_RAT	U		
FOX2	gi-13242303	alpha	peroxisomal multifunctional beta-oxidation protein
gi-4105269	alpha	putative peroxisomal 2,4-dienoyl-CoA reductase	
gi-5052204	alpha	putative short-chain dehydrogenase/reductase	
FOX3	gi-6978429	U	peroxisomal 3-oxoacyl CoA thiolase
-	ECHP_RAT	U	Peroxisomal bifunctional enzyme
-	SCP2	U	sterol carrier protein-2
IDP3	gi-13928690	U	Peroxisomal NADP-dependent isocitrate dehydrogenase
ECI1	gi-6755026	alpha	enoyl-CoA isomerase
DCI1	alpha		
-	BAAT	alpha	bile acid-Coenzyme A: amino acid N-acyltransferase
-	gi-12002203	actinomyc.	alkyl-dihydroxyacetonephosphate synthase
-	DAPT_RAT	actinomyc.	Dihydroxyacetone phosphate acyltransferase
-	AGT	cyanobac.	alanine-glyoxylate aminotransferase
-	gi-6679507	U	pipecolic acid oxidase
-	URIC_RAT	U	Urate oxidase
PXA1	PMP70	U	fatty acid transport
ALDP	U	ATP-binding cassette	
ALDPR	U	ATP-binding cassette	
FAA1	-	euk	
FAA2	LCF2	U	Long-chain-fatty-acid--CoA ligase
LACS	U		
FAT1	VLACS	U	Fatty acid transport
-	gi-14091775	U	Hydroxyacid oxidase 3 (medium-chain)
-	gi-6754156	U	Hydroxyacid oxidase 1
-	GTK1_RAT	U	Glutathyhion-S transferase
-	AMCR	U	2-arylpropionyl-CoA epimerase
-	FIS1	euk	Peroxisome fission
FAT2	-	alpha	probable AMP-binding protein
CIT2	-	U	Citrate synthase
GPD1	-	alpha	glycerol-3-phosphate dehydrogenase
MDH3	-	U	malate dehydrogenase
LYS1	-	euk	Lysine biosynthesis, saccharopine dehydrogenase
LYS4	-	U	Lysine biosynthesis
PNC1	-	U	NAD(+) salvage pathway
NPY1	-	alpha	NADH diphosphatase (pyrophosphatase)
STR3	-	U	Sulfur Transfer
YGR154C	-	U	
MLS1	-	U	Malate synthase 1
MLS2	-	U	Malate synthase 2
EMP24	-	euk	Vesicle organization and biogenesis
ERG1	-	U	Ergosterol biosynthesis
ERG6	-	U	Ergosterol biosynthesis
RHO1	-	euk	GTP-binding protein
SPS19	-	U	2,4-dienoyl-CoA reductase
YOR084W	-	euk	Peroxisome organization and biogenesis
YMR204C	-	euk	
CAT2	-	euk	Carnitine acetyltransferase
PCD1	-	alpha	Nudix hydrolase
AAT2	-	euk	Aspartate aminotransferase
PXA2	-	U	Peroxisomal ATP-binding cassette, fatty acid transport
VPS1	-	euk	Dynamin 1

### Peroxisomal proteins of eukaryotic origin and an evolutionary link with the E.R

**Figure 2:**
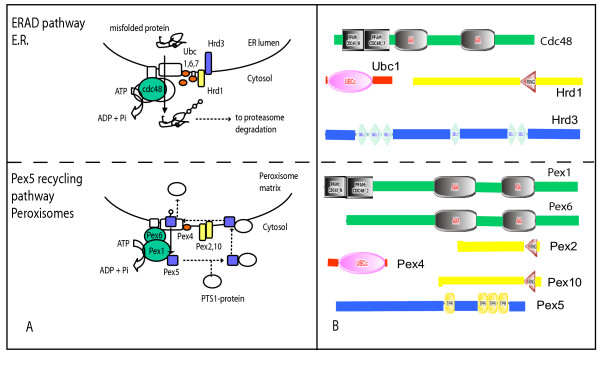
ERAD and peroxisomal protein import homology. A) Schematic representation of the ERAD (top) and the Pex5 recycling (bottom) pathways. Proteins involved are represented by ovals and rectangles, only those commented in the text are named. Homologous relationships between proteins from the pathways are indicated in color. B) Homology between proteins of the ERAD pathway and proteins involved in protein import to the Peroxisome. Domain organization of the proteins was predicted with SMART [45]. Independent from that, homology between the proteins was determined by profile-to-profile searches using hhsearch [46], based on alignments of orthologous groups of the various proteins. Note that the SEL1 repeat is homologous to the TPR repeat. The location of the two CDC48 N-terminal domains (CDC48_N and CDC48_2) in Pex1 is based on PSI-Blast [47] searches starting with CDC48 proteins and on the structure published for the N-terminal domains of PEX1 [48].

The largest fraction of peroxisomal proteins is of eukaryotic origin: 58.1% of the yeast proteome, 39.2% of the rat proteome (Figure 1c). These include the so-called Pex proteins that are involved in peroxisomal biogenesis and maintenance that are most consistently present in all microbodies, underlining their essential role. Interestingly, five of the six most ancient Pex proteins (see below) show homology with the ERAD (Endoplasmic Reticulum Associated Decay) system, which pulls proteins from the ER membrane and ubiquitinylates them in preparation for degradation in the proteasome [12] (Figure 2). Pex1 and Pex6, AAA cassette containing proteins, have evolved from Cdc48/p97 [13] (Figure 1a), a protein central to the ERAD pathway which is also involved in Golgi vesicle fusion and spindle body disassembly after mitosis; Pex2 and Pex10, ubiquitin ligase domain (RING domain) containing proteins, contain homology to the ERAD ubiquitin ligase Hrd1; the TPR repeats of Pex5 are homologous to the SEL1 repeats of the Hrd1 interacting protein Hrd3; Pex4 contains an E2 ubiquitin conjugating enzyme domain and is homologous to the ERAD ubiquitin conjugating enzymes Ubc1, Ubc6 and Ubc7. In the cases of PEX2/10, PEX5 and PEX4 the levels of sequence identity between the shared domains and the short regions of homology preclude the reconstruction of reliable phylogenies to argue that these proteins have descended from a protein involved in ERAD, as it is the case for Cdc48/p97-PEX1/6. Here it is the number of homologous relations between ERAD and the most ancient PEX proteins that hint at an evolutionary relation. Although there are some systems known that use a TPR repeat protein together with a protein containing an E2 ubiquitin conjugating enzyme and a protein with a RING domain, like the Anaphase Promoting Complex/Cyclosome [14], to our knowledge there is no system other than ERAD that uses those domains together with an AAA+ ATPase. Nevertheless, we cannot exclude that PEX1, PEX2/10, PEX5 and PEX4 do not originate from a single molecular system like ERAD, specifically because the TPR repeat in HRD3 is classified in a different class of TPR repeats than the TPR repeat of PEX5 (Figure 2).

The similarities in amino acid sequence between ERAD and the most ancient PEX proteins extend into similarities in function and sub-cellular location (Figure 2). Pex1 and Pex6 (both AAA containing proteins) are needed to extract the cycling PTS1 receptor Pex5 from the peroxisomal membrane to facilitate a new cycle of Pex5-mediated protein import [15]. Ubiquitinylation of Pex5 is part of this process. In both cases, the ERAD and the peroxisomal AAA proteins operate in the cytoplasm and are recruited to the membrane by organelle-specific anchor proteins: Cdc48/p97 to the ER membrane by VIMP[16], Pex1 and Pex6 to the peroxisomal membrane by Pex15 (in yeast) and Pex26 (in mammals) [17]. This resemblance in ancient proteins with similar functions and the link with the universal endomembrane compartment of the eukaryotic cell suggest that the peroxisome is an invention that took place within the eukaryotic lineage itself. Also Erdmann and Schliebs [18] have recently linked the homology between AAA+ domains of ERAD and PEX1, and the presence of E2 and E3 domains involved in ubiquitinylation in the PEX proteins, to a mechanism of protein import in the Peroxisomal matrix that would be similar to ERAD, without proposing a direct evolutionary descent of Peroxisomal import from ERAD however.

For the other PEX proteins we did not find indications that they were also recruited from pre-existing cellular systems. Their distribution and phylogenies do suggest that they originate from separate events post-dating the origin of the five of the six core PEX proteins from ERAD.

**Figure 3:**
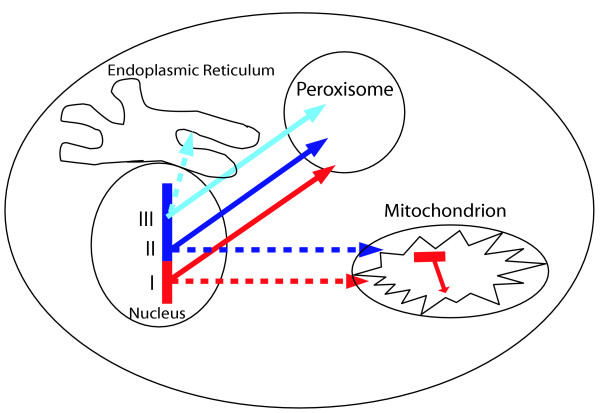
The retargeting of proteins to the peroxisome during evolution. The dashed lines indicate the ancestral cellular location of a peroxisomal protein, the continuous line their current (peroxisomal) location. Some proteins are derived from the alpha-proteobacterial ancestor of the mitochondria, their proteins have been retargeted to the peroxisome concomitant with the transfer of their genes to the nucleus (red, scenario I). Also proteins without a (detectable) alpha-proteobacterial ancestry have been retargeted from the mitochondria (blue, scenario II). Finally, a class of proteins have been retargeted from other compartments of the cell like the Endoplasmic Reticulum (cyan, scenario III).

We have visualized the retargeting during evolution of peroxisomal proteins from various cellular locations in a cartoon (Figure 3). The group of proteins of eukaryotic origin also contains certain household proteins with dual or plural functions with respect to organelles. The ER located or associated proteins Erg1, Erg6, Emp24, Rho1 and the multipurpose dynamin Vps1 have also been implicated in peroxisomal functions [19,20].

### Recruitment to the peroxisome of proteins of alpha-proteobacterial origin

Remarkably, the second largest fraction of proteins, 17–18%, has an alpha-proteobacterial origin (Figure 1c). This is similar to what has been found for mitochondria [7,8], and, at first sight appears to be at odds with a eukaryotic origin of the peroxisome. There are strong indications that these proteins have been retargeted from the mitochondria (Figure 3, scenario I), rather than having evolved directly from an independent endosymbiont, an observation that is consistent with the high degree of retargeting observed for proteins derived from the proto-mitochondrion in general [8]. Six of the eight *S. cerevisiae *peroxisomal proteins of alpha-proteobacterial origin are closely related to mitochondrial proteins. Thioesterase (Tes1p) is located in both the peroxisome and mitochondrion of *S. cerevisiae *[21]. In other cases the orthologs or paralogs of a peroxisomal protein are mitochondrial: i), the peroxisomal glycerole-P dehydrogenase Gpd1p has a paralog in yeast (Gpd2p) with a cytoplasmic and mitochondrial localization [22]; ii) the peroxisomal Fat2p is paralogous to the mitochondrial long-chain fatty acid CoA ligases iii), the orthologous group consisting of Eci1p, Dci1p and 3,2-transenoyl CoA isomerase is peroxisomal in yeast and human, has a mitochondrial paralog in mammals [23]; and iv), the nudix phosphatase family (Npy1p) of which the yeast, human and plant orthologs are peroxisomal has a paralogous group in metazoa that is mitochondrial according to GFP-fusion studies in mouse [24] and to Mitoprot [25] (p = 0.97). The phylogenetic tree (Figure 1b) indicates a single origin from the alpha-proteobacteria of both mitochondrial and peroxisomal proteins of this family. The two remaining cases of yeast peroxisomal proteins of alpha-proteobacterial origin are Fox2p and Pcd1p. For these no homologs with experimental evidence of mitochondrial location were found, although Pcd1p does have a bona-fide mitochondrial targeting signal (P = 0.97 in Mitoprot).

With respect to the rat peroxisome, there are two proteins of alpha-proteobacterial descent that do not have orthologs in the yeast peroxisome. One of these presents cases of dual targeting: some isoforms of peroxisomal bile acid thioestherase BAAT have been detected in mitochondria and the cytoplasm in human liver [26].

### Recruitment to the peroxisome of mitochondrial proteins of other origins

**Figure 4:**
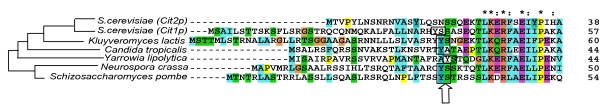
The N-terminal region of the multiple sequence alignment of several fungal members of the Cit1/2p orthologous group. Amino acids around the signal-peptide cleavage-sites, as predicted by Mitoprot are marked with a rectangle (white arrow indicates the position in the alignment) they correspond to YS (YA in *C. tropicalis*) that is missing in Cit2p. No mitochondrial localization nor a cleavage-site is predicted for Cit2p consistent with its peroxisomal location.

There are also peroxisomal proteins with homologs in the mitochondrion that do not have a (detectable) alpha-proteobacterial origin: Idp3p, Cta1p, Faa1p, Cit2p, Fis1p and Faa2p [21,27,28] (Figure 3, Scenario II). In contrast to proteins of alpha-proteobacterial origin, here one cannot simply argue that the mitochondrial localization preceded the peroxisomal one. At least for one of these proteins, Cit2p, a peroxisomal protein from the citrate synthase family, a phylogenetic analysis reveals its ancestral location. The other two members of this family in *S. cerevisiae*, Cit1p and Cit3p, are mitochondrial and so are their homologs in *Homo sapiens*, *Arabidopsis thaliana *and *Caenorhabditis elegans*. The phylogeny of this family in fungi indicates that Cit1p and Cit2p originate from a recent gene duplication, after which Cit2p lost its mitochondrial targeting signal (Figure 4), indicating that the peroxisomal location is secondary. That the retargeting of proteins between mitochondria and peroxisomes frequently happens during evolution is also indicated by the case of alanine:glyoxylate aminotransferase (AGT), whose peroxisomal or mitochondrial location is species-dependent and related to diet in mammals [29]. In humans, where AGT is peroxisomal, a single point mutation miss-localizes the protein to the mitochondrion, leading to the hereditary kidney stone disease: primary hyperoxaluria type 1 (PH1)[30].

There are peroxisomal rat proteins, like dihydroxyacetone phosphate acyl transferase(DAPT) and alkyl-dihydroxyacetonephosphate synthase (gi-12002203) whose phylogenetic trees suggest an ancestry from within the actinomycetales while the Alanine-Glyoxylate aminotransferase (AGT) appears derived from the cyanobacteria. We do not have an obvious evolutionary scenario for the origin for such proteins with a bacterial but not alpha-proteobacterial ancestry. In any case, the finding of peroxisomal proteins with such diverse origins underscores the ease at which the peroxisomal proteome can recruit new proteins.

### Reconstruction of ancestral states of the peroxisomal proteome

**Figure 5:**
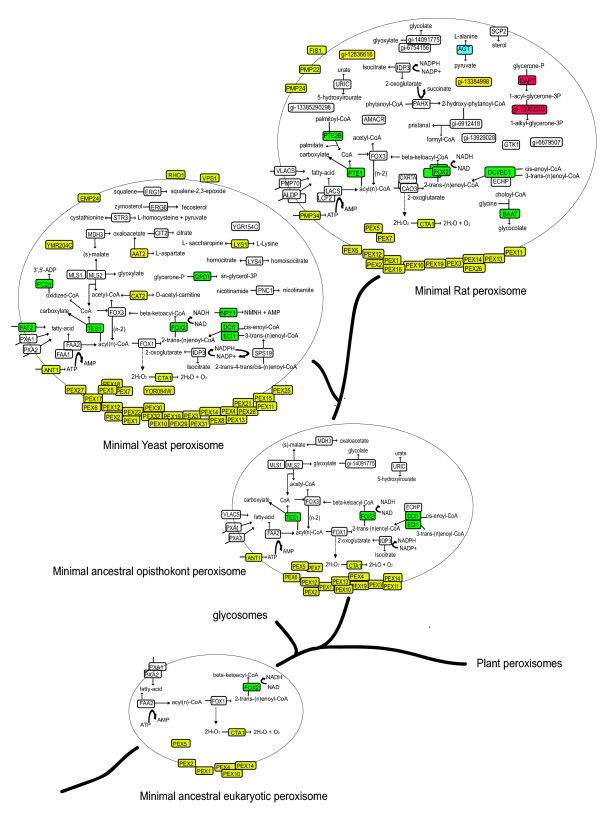
Evolution of the peroxisomal proteome. Biochemical pathways reconstructed according to KEGG and annotations of peroxisomal proteins. For details on the reconstruction of ancestral states see supplemental material. Color code: yellow, eukaryotic origin; green, alpha-proteobacterial origin; red, actinomycetales origin; blue, cyanobacterial origin; white, origin unresolved. Note that the ancestral eukaryotic peroxisomal proteome reconstruction depends on the topology of the eukaryotic tree. If an alternative topology is considered, placing kinetoplastida and viridiplantae together [49], and the plant peroxisomal proteome is taken from the Araperox database [31], then the reconstructed ancestral eukaryotic peroxisomal proteome would be much larger, including all proteins present in the opisthokont proteome except for ANT1, IDP3, FOX3, PEX13 and PEX19.

To investigate the order of protein recruitment to the peroxisome we reconstructed the evolution of the peroxisomal proteome based on the absence/presence of genes among sequenced genomes and assuming a parsimonious scenario (Figure 5). First we reconstructed the minimal peroxisome of the opisthokont, the common ancestor of metazoa and fungi, by including proteins present in both yeast and rat peroxisomal proteomes or proteins that are present in only one of the two proteomes but whose orthologs in plants have a (putative) peroxisomal location in the Araperox database [31]. In addition, we reconstructed the protein content of the common ancestor of all known peroxisomes, glycosomes and glyoxysomes from proteins that, besides being present in the opisthokont peroxisome, are present in genomes from plants and kinetoplastida (*Trypanosoma brucei *and *Leishmania major*). This core-set comprises six PEX proteins (Pex1p, Pex2p, Pex4p, Pex5p, Pex10p, Pex14p) and four proteins involved in fatty acid metabolism and transport (Pxa1p/Pxa2p, Fox2p, Faa2p). We also included the peroxisomal hallmark protein catalase (Cta1p), even though it is absent from most glycosomes and kinetoplastidial genomes because it is found in the glycosomes of the non-pathogenic trypanosomatid *Crithidia *[32]. Similarly Fox1p, which catalyzes the first step of long-chain fatty acid beta-oxidation, was included despite its absence from kinetoplastida, because the concomitant loss from peroxisomes of Fox1 (the enzyme generating H_2_O_2_) and catalase (the enzyme detoxifying H_2_O_2_) has been observed in species such as *Neurospora crassa *[33].

Although the specific functional role of many PEX proteins remains to be established, and it is therewith hard to asses whether e.g. the reconstructed ancestral opisthokont PEX proteins are functionally coherent and complete, at least the sub-set present in the ancestral eukaryotic peroxisome appears functionally coherent. All of the six universal PEX proteins are specifically involved in the PEX5 pathway for the import of proteins into the peroxisome.

The earliest tractable function of peroxisomes appears herewith to be the beta-oxidation of fatty acids. This pathway already contains at least one protein of alpha-proteobacterial descent (Fox2p), indicating that the presence of long-chain fatty acid beta-oxidation in the peroxisome followed the endosymbiosis of mitochondria. The proteins with detectable origin in the ancestral peroxisome that are not involved in beta-oxidation are all of eukaryotic origin. Most of the present-day species variability is found in the enzymes housed in peroxisomes, a significant fraction of which has an alpha-proteobacterial origin and has entered the primitive eukaryote with the mitochondrial ancestor [8]. Note that the recruitment of proteins with an endosymbiotic origin to peroxisomes is not an exceptional event. Nine proteins in the glycosomes of the kinetoplastida *T. brucei *and *Leishmania mexicana *are derived from chloroplasts from which they can be traced back to the cyanobacteria [34]. Somehow it seems rather easy to (re)locate proteins to microbodies which may be related to the simplicity of the PTS1 targeting code. This 'grab what you can get' principle may have contributed to the observed versatility and species variability.

## Conclusion

The phylogenetic analysis of the rat and yeast peroxisomal proteomes reveals that the largest fraction of peroxisomal proteins originated within the eukaryotic lineage and that the significant fraction of peroxisomal proteins which stems from the alpha-proteobacteria is likely the result of a secondary retargeting from the mitochondrion. The most widespread and ancient set of peroxisomal proteins is mainly composed of eukaryotic proteins involved in peroxisome biogenesis and organization. Most of these core proteins are evolutionarily related to the Endoplasmic Reticulum Assisted Decay pathway, suggesting an evolutionary origin of the peroxisomes from the endomembrane system. While this manuscript was under review a common evolutionary origin of the Peroxisome and the ER was also proposed by Schluter and coworkers [35] based on the homologies in Figure 2 between ERAD and Pex proteins (Figure 2) while this homology has also been observed by Erdman and Schliebs [18]. In the former analysis, full length homologs with Bacterial proteins were not included and the authors could not exclude that such proteins were indeed donated by an early symbiont. The retargeting of enzymes documented in this paper solves the paradox of a eukaryotic organelle with bacterial enzymes. Recent experimental work indicates that some peroxisomal proteins first enter the ER thereby capturing part of the ER membrane for subsequent formation of the organelle [6]. These observations are consistent with our findings that the oldest PEX proteins are homologous to proteins of the ERAD pathway, suggesting that evolutionarily as well as ontogenetically peroxisomes are in fact offshoots from the ER.

## Methods

### Data retrieval

Manually curated sets of 62 *S. cerevisiae *and 50 *R. norvegicus *proteins with experimental evidence of peroxisomal location were compiled from the literature [9-11,19] and from the Saccharomyces Genome [36] and Swiss-Prot [37] databases. For the purpose of this paper we consider a protein to be peroxisomal when it permanently resides in the peroxisomal matrix or membrane, or when it is a cytoplasmic protein but has a dedicated function in peroxisomal protein import and/or biogenesis.

Protein sequences encoded by 144 publicly available complete genomes were obtained from Swissprot [37], except for *Plasmodium falciparum, Schizosaccharomyce pombe, Candida albicans, Encephalitozoon cuniculi *(Genbank, [38]), *Homo sapiens, Rattus norvegicus *and *Mus musculus *(EBI, [39]).

### Phylogenetic reconstructions

For every yeast and rat peroxisomal protein, homologous sequences (E < 0.01) were retrieved using Smith-Waterman comparisons against the aforementioned 144 complete proteomes. Only sequences that aligned with at least one third of the query sequence were selected. Sequences were aligned using MUSCLE [40]. Neighbour Joining (NJ) trees were made using Kimura distances as implemented in ClustalW [41]. Positions with gaps were excluded and 1000 bootstrap iterations were performed. Maximum Likelihood (ML) trees were derived using PhyML v2.1b1 [42], with a four rate gamma-distribution model, before and after excluding from the alignment positions with gaps in 10% or more of the sequences. In all cases NJ and ML trees were manually examined to search for consistent patterns indicating the origin of the peroxisomal proteins. Trees in which eukaryotic proteins clustered together, within the Bacteria or the Archaea and with a specific prokaryotic out-group were classified as having that phylogenetic origin (e.g. Figure 1b). Trees were only regarded as resolved when both the NJ tree and the ML tree agreed to the level of resolution required, e.g. a specific bacterial group as a sister clade of the peroxisomal group of proteins, or when at least the ML tree had the level of resolution required while the NJ tree did not point to another origin of a protein.

### Reconstruction of yeast, rat peroxisomal metabolisms and their ancestral states

Annotated biochemical and cellular functions of the yeast and rat peroxisomal proteins were mapped onto metabolic KEGG maps [43] and are represented in Figure 5, indicating their phylogenetic origin by a color-code. Proteins known or predicted to be membrane-associated are depicted close to the membrane. The minimal ancestral opisthokont peroxisome was reconstructed by combining proteins that are present in both yeast and rat peroxisomal proteomes or that are present in only one of the two proteomes but have orthologs in plants with a peroxisomal location or are described as putative peroxisomal proteins in Araperox database [31]. The minimal ancestral eukaryotic proteome is formed by those proteins of the ancestral opisthokont proteome that are also found in the genomes of plants, *Typanosoma brucei *and *Leishmania major*. Catalase and Fox1 that are absent from glycosomes were nevertheless included for the reasons explained in the results and discussion section.

## Authors' contributions

TG and MAH conceived the study, participated in its design and coordination. TG, MAH, BS and FvZ participated in the computational analysis of peroxisomal proteins. TG, MAH, BS and HT drafted the manuscript. WH provided important insights in the study and the results. All authors read and approved the final manuscript.

## Reviewers' comments

### Reviewer's report 1

Arcady Mushegian. Bioinformatics center. Stowers institute for medical research. Kansas City. Missouri. USA.

1. I suggest that the relationship with ERAD is addressed further, e.g. by including ERAD components into Table 1 and by adding detail to Figure 2A.

Response:

We now indicate in the table those peroxisomal proteins that show homology with components of the ERAD pathway. In order to provide more detail to figure 2.A we have included the role of ubiquitine in both the processes of ERAD and peroxisomal import. Moreover we have re-arranged the proteins and arrows so that their mechanism of action is clearer. Nevertheless the exact functioning of either ERAD or the PEX5 pathway for protein import has not completely been resolved.

2. What is the identity of actinomycete-like, cyanobacteria-like, and "unresolved" components of peroxisome in Figure 1? What is the explanation for the existence of the first two groups? What can be said about functions of "resolved" vs "unresolved" groups – any trends there?

Response:

The identity of the actinomycete-like and cyanobacteria-like proteins is indicated now in the text, they can also be seen in Figure 3 and the table. We do not have a plausible explanation for the origin of these proteins and that is now mentioned in the text. We discuss as well the observed functional dichotomy observed in the proteins with prokaryotic or eukaryotic ancestry for the resolved cases as well as the preponderance of enzymes in the unresolved cases.

3. On the ancestral reconstruction: which parsimony was used – unweighted or weighted? Are opisthocont and eukaryotic sets of PEX genes functionally coherent, or are there missing components?

Response:

We used a simple parsimony approach in deciding where certain proteins appeared in evolution: a protein is supposed to have been present at the root of the smallest partition containing all genomes that have that gene. The functional coherence of the PEX subsets is difficult to assess, since many PEX proteins have no specific function assigned. However, at least for the ancestral eukaryotic peroxisome the subset of PEX proteins recovered are all involved in peroxisomal protein import, as indicated in the text.

### Reviewer's report 2

Gáspár Jékely. European Molecular Biology Laboratory. Heidelberg, Germany.

This paper makes a compelling argument for the autogenous evolutionary origin of the peroxisome. Although this was not a surprise given recent cell biological findings showing that peroxisomes grow from the endoplasmic reticulum, the autogenous origin of the organelle is now clearly backed by the systematic bioinformatic analysis of its proteome. Most interestingly Gabaldón et al. found that some components of the peroxisomal proteome (the Pex5 pathway) are evolutionary derivatives of the endoplasmic reticulum assisted decay (ERAD) pathway.

The paper is technically sound and well written, I only have a few comments.

1) I have a problem about how the authors define that a protein has eukaryotic origin. For example the Cdc48/Sec18/Pex6 family seems to have descended from archaebacterial AAA ATPases. What the tree shown in Fig. 1A shows is rather that the multiplication of this ancestral ATPase leading to several paralogs was an eukaryotic event. So the protein family clearly has prokaryotic origin, it is the formation of distinct paralogs that occurred during eukaryote evolution. This should be explained better in the text and this group should be referred to differently, like 'originated by eukaryote-specific duplication'.

Response:

We specifically want to make a distinction between horizontally transferred genes and "ancient genes" that were already present at the evolutionary split between the lineage leading to the Archaea and the one leading to the eukaryotes. Although in the case of CDC48 and Pex1 a case can indeed be made that CDC48 represents the ancestral function, given its level of sequence identity with the Archaeal sequences, and that PEX1 resulted from a gene duplication, such a clear scenario is rarely present. We have put more emphasis on the distinction between horizontally transferred genes and genes already present in ancient eukaryotes in the text, and mention the CDC48 duplication explicitly now.

2) The reconstruction of the ancestral state of the peroxisomal proteome hinges on the accepted topology of the eukaryotic tree. If Kinetoplastids are not early branching but the root lies between animals and plants, then one would probably get a different picture. This alternative reconstruction should also be presented and/or the effect of tree topology on the results should be discussed.

Response:

The consequences of using an alternative topology in the reconstruction of the ancestral proteome are now mentioned in the figure legend. They indeed lead to a larger set of ancestral Peroxisomal proteins.

3) Several of the eukaryote-specific Pex proteins are not discussed in the text. One is left wondering what could have been the evolutionary origin of these proteins. If it is not clear for most of them, this should be mentioned briefly.

Response:

We tried hard to ascertain the origin of all Pex proteins, unfortunately for the Pex proteins not discussed in the text we could not find homologies with other proteins of known function or these were too weak to be considered reliable. We now explicitly mention this fact.

### Reviewer's report 3

John M. Logsdon, Jr., Department of Biological Sciences, Roy J. Carver Center for Comparative Genomics, University of Iowa, Iowa City, IA 52242 USA

Comments:

This paper reports the "phylogenomic" analysis of peroxisomal proteins with an aim to distinguish between an endosymbiotic vs. endogenous origin of this organelle in eukaryotic cells. This has been a long-standing question in the evolution of eukaryotic cells and these authors have provided a compelling analysis that rejects the hypothesis that the peroxisome is of endosymbiotic origin. Instead, the data indicate an endogenous origin of peroxisomes from the endoplasmic reticulum.

The authors compiled a curated set of peroxisomal proteins from two major model systems in which global proteomic studies of the peroxisome have been done: yeast and rat. These protein sets, thus, represent a large fraction of the peroxisomal proteome. The authors then used a systematic and rigorous analysis procedure to identify all of the homologs of these proteins from among available complete genomes (prokaryotic and eukaryotic). For all peroxisomal proteins and their homologs, phylogenetic trees were reconstructed and the topologies were evaluated to determine the evolutionary history of each peroxisomal gene. The analysis methodology used is appropriately robust to the questions asked.

1) Although I wholly recommend the publication of this work in Biology Direct, it should be noted that, during the process of review, another paper reporting the same conclusions has appeared as an "Advance Access" publication at Molecular Biology and Evolution:

A. Schlüter, R. Ripp, S. Fourcade, J. L. Mandel, O. Poch, A. Pujol, "The Evolutionary Origin of Peroxisomes: An ER-Peroxisome Connection". I am satisfied that the approaches taken here are sufficiently different than those used by Schlüter et al. and thus merit separate publication. However, it would be helpful for the authors here to refer to the Schlüter et al. paper in their revision and to compare and contrast their approaches and results if at all possible. In addition, it is suggested that the authors consider changing their title so as to not so closely resemble the Schlüter et al. paper.

Response:

The Schlüter paper addresses the origin of Peroxisomal proteins without bacterial homologs. As can be seen from our analysis there is actually a conflict in the conclusion one can draw from on the one hand the presence of proteins with alpha-proteobacterial ancestry and on the other hand from the presence of proteins with ER ancestry. One can only resolve this by addressing the retargeting of proteins with alpha-proteobacterial ancestry as we have done in our analysis. We explicitly refer to the Schlüter paper and its observation of the link with the ER in the conclusion, and have changed the title of our paper.

2) Figure 3, and the verbiage associated with it (last paragraph of "Peroxisomal proteins..." section), is confusing and should either be clarified (expanded) or deleted. The figure seems too abstract to be useful. What do the dashed arrows mean?

Response:

Figure 3 depicts the moving of DNA and protein localization in evolution, which is rather central to the manuscript. We have rephrased the legend, including an explanation of the meaning of the dashed arrows.

3) Figure 4 is unnecessary to the main message of the paper and could instead be included as a supplement. In fact, it would seem that the phylogeny of this gene family would be a more relevant figure, given the verbiage in the manuscript.

Response

We have left Figure 4 in. It includes the most relevant part of the phylogeny of the citrate synthase genes and does illustrate how the retargeting of proteins has continued in recent evolution and is even visible in the sequences.

4) The phylogenetic trees that are provided as supplementary data are supplied as a single webpage  with the trees given in newick format. Although providing these data in a supplemental format is perfectly acceptable, the authors should provide graphic versions of each tree. Indeed, the abbreviations used for the sequences/taxa in these trees are apparently not defined anywhere in the manuscript. Thus, a key to the taxa is at a minimum required, but even better would be a clear labeling of all of the taxon names on all of trees.

Response:

The taxonomic names of the species and the trees are being included.
